# Validity Evidence for the Secondary Symptoms of the Burnout Assessment Tool: A Brazilian Study

**DOI:** 10.3390/ijerph23030302

**Published:** 2026-02-28

**Authors:** Andrea Marilin Vinueza-Solórzano, Jaqueline de Carvalho Rodrigues, Clarissa Pinto Pizarro de Freitas, Wilmar B. Schaufeli, Hans De Witte, Ana Claudia Souza Vazquez, Cecilia Alexandra Portalanza-Chavarria

**Affiliations:** 1 Department of Psychology, Pontifícia Universidade Católica de Rio de Janeiro, Rio de Janeiro 22451-900, RJ, Brazil; jaquelinerodrigues@puc-rio.br; 2 Department of Psychology, Pontificia Universidade Católica de Rio Grande do Sul, Porto Alegre 90619-900, RS, Brazil; clarissa.freitas@pucrs.br; 3 Research Group Work, Organizational and Personnel Psychology, KU Leuven, 3000 Leuven, Belgiumhans.dewitte@kuleuven.be (H.D.W.); 4 Department of Social and Organizational Psychology, Utrecht University, 3584 CS Utrecht, The Netherlands; 5 Optentia Research Unit, North-West University, Vanderbijlpark 1900, South Africa; 6 Department of Psychology, Universidade Federal de Ciências da Saúde de Porto Alegre, Porto Alegre 90050-170, RS, Brazil; anasv@ufcspa.edu.br; 7 Research Center, University Espiritu Santo, Samborondón 09-01-952, Ecuador; aportalanza@uees.edu.ec

**Keywords:** burnout, secondary symptoms, validity, reliability

## Abstract

**Highlights:**

**Public health relevance—How does this work relate to a public health issue?**

- This study addresses burnout as a multidimensional occupational health condition, as conceptualized by the Burnout Assessment Tool (BAT), encompassing exhaustion, mental distancing, cognitive impairment and emotional impairment, complemented with psychological distress and psychosomatic complaints as secondary symptoms of burnout.
- By applying the BAT framework, the study contributes to improved identification and monitoring of burnout symptoms that represent a growing public mental health concern among working populations.

**Public health significance—Why is this work of significance to public health?**

- The use of the Burnout Assessment Tool (BAT) provides a theoretically grounded and comprehensive assessment of burnout, supporting more accurate estimation of burnout-related mental health risks.
- The findings strengthen the evidence base for recognizing burnout as a relevant indicator of psychological and psychosomatic functioning with implications for mental health surveillance.

**Public health implications—What are the key implications or messages for practitioners, policy makers and/or researchers in public health?**

- The results support the use of the BAT as a standardized instrument for assessing burnout symptoms in occupational and public health contexts.
- The study highlights the importance of early detection of core and secondary burnout symptoms to inform preventive strategies and guide mental health promotion initiatives in the workplace.

**Abstract:**

Burnout syndrome is conceptualized as a work-related psychological condition primarily marked by persistent exhaustion, emotional and cognitive impairment and mental distancing. In addition to these core dimensions, burnout may give rise to secondary symptoms, including psychological distress, psychosomatic complaints, and depressive mood. The Burnout Assessment Tool (BAT) includes specific measures for both primary and secondary symptoms. This study aimed to evaluate the validity evidence of the BAT’s secondary symptoms Brazilian version scale (BAT-S). The sample consisted of 1.750 professionals (71% women), with a mean age of 39 years (SD = 11). Confirmatory Factor Analyses indicated that a model of two oblique first-order factors, differentiating psychological distress from psychosomatic complaints, provided a superior fit compared to the unidimensional solution. The scale also presented satisfactory internal consistency for the scales of psychological distress (α = 0.88 and ω = 0.90) and psychosomatic complaints (α = 0.85 and ω = 0.87). The BAT-S represents a reliable tool to assess these secondary symptoms of burnout, advancing research that integrates behavioral and physiological markers, offering practical applications for occupational health interventions and preventive strategies in the workplace.

## 1. Introduction

A rising body of studies on burnout syndrome has made it a well-known term, recently categorized as a work-related disorder in the ICD-11 [1]. Although it has been studied since the 1970s, it is still a subject of conceptual debate; therefore, it is considered a challenge for academics in areas such as organizational psychology and occupational health psychology [2]. Furthermore, its accurate assessment is vital for identifying at-risk individuals and informing organizational interventions aimed at promoting mental health and sustainable work conditions. During the COVID-19 pandemic, occupational health has gained unprecedented importance [3,4], highlighting burnout as a critical phenomenon that organizational decision-makers worldwide seek to understand better.

Burnout is defined as a state of mental exhaustion and is an occupational phenomenon that results from chronic stress in the workplace [1]. The traditional approach to burnout, mainly influenced by the Maslach Burnout Inventory (MBI), characterizes the syndrome through three dimensions: emotional exhaustion, depersonalization, and reduced capacity (occupational overload) [5]. Even though the validity and reliability of the MBI questionnaires have been statistically validated in multiple studies, there are still conceptual, psychometric, and practical shortcomings [5,6]. These limitations led to develop the Burnout Assessment Tool (BAT), as an alternative instrument that would overcome the limitations identified in MBI. The latter is criticized for emphasizing emotional exhaustion while neglecting other dimensions like cognitive processes, for the lack of a general burnout score that shows the level of burnout developed and for conflating burnout with stress or depression [6]. BAT addresses these limitations by incorporating both core and secondary symptoms into a multidimensional framework, thereby offering a more comprehensive and clinically relevant assessment approach [7]. The present study searches for theoretical implications for burnout conceptualization and assessment. This study aims to explore the theoretical implications for the conceptualization and assessment of burnout. Burnout research has long faced criticism regarding theoretical fragility, measurement issues, and limited clinical utility [8].

The BAT was developed by applying an inductive and deductive approach to select the items that constitute the instrument [7]. An important feature of the BAT is the assessment of burnout as a state with a total score, complementing the evaluation of its constituent dimensions. The complete work-related version of the BAT contains 33 items and consists of the BAT-C for the core symptoms and BAT-S for secondary symptoms. The BAT-C assesses the four core dimensions (exhaustion, mental distance, emotional impairment, and cognitive impairment) and contains 23 items. These core symptoms can be assessed using either a long-form version of the instrument, consisting of 23 items, or a short-form version comprising 12 items; there is even the ultra-short 4-item version already validated [9]. The BAT-S assesses the two secondary dimensions: psychological distress (e.g., sleep problems, tension, and worrying) and psychosomatic complaints (e.g., headaches, chest, and muscle pain) and contains 10 items. Both are rated on a five-point Likert scale ranging from never (1) to always (5) [7].

The BAT-C has been adapted and validated in multiple countries [10], such as: Brazil [11], Croatia [12], Ecuador [13], Finland [14], Greece [15], Italy [16,17,18], Japan [19], Korea [20], Lithuania [21], Netherlands [22], Poland [23], Portugal [24], Romania [25], South Africa [26] and Turkey [27]. Moreover, studies have demonstrated that the BAT’s second-order factor structure remains invariant across countries, suggesting its utility for reliable and valid cross-national comparisons [24,26].

The BAT’s conceptualization of burnout as a syndrome needs the generation of both a composite score that represents the overall burnout syndrome and individual scores for each of the four core symptoms. To evaluate the structure of the BAT, researchers have compared a four-factor model, in which the BAT-C consists of four correlated subscales, with a second-order model, where all subscales load onto a higher-order burnout factor. Empirical evidence supports the superiority of the second-order model, which aligns with the syndrome conceptualization of burnout, over the four-dimensional model [28]. The internal consistency of the BAT-C has also been extensively tested, with results indicating high reliability (exceeding 0.80 for the subscales and 0.90 for the overall scale) across various countries, including Austria, the Netherlands, Ireland, Belgium (Flanders), Finland, Germany [29], Japan [19], Italy [16], Ecuador [13], Korea [20], Brazil [11], Portugal [24], and Romania [25].

In terms of discriminant validity, research has demonstrated that the BAT is distinct from related constructs, such as job boredom, depressed mood, workaholism, and work engagement [7]. These results were also replicated in a Romanian sample evaluating burnout through BAT-12 [25]. In a Brazilian sample, burnout assessed through BAT-12 showed discriminant validity with respect to depressive symptoms, anxiety, and irritability, indicating that BAT scores captured variance distinct from these affective domains [30]. Additionally, it was alsofound positive correlations of burnout with workaholism and negative with work engagement measured by the BAT-C of 12 items, both directly and indirectly through self-endangering behavior [19].

In the BAT research, the secondary dimensions are described as follows: (1) psychological distress, which encompasses unpleasant emotions associated with heightened arousal that negatively impact functioning and interfere with daily activities; (2) psychosomatic complaints, referring to physical symptoms that are believed to be caused or aggravated by psychological factors; (3) depressed mood, which, while distinct from major depressive disorder or other mood disorders, represents a common emotional response to disappointment rather than a psychiatric disorder [7]. These three symptoms are considered atypical for burnout as they theoretically do not reflect either an inability or unwillingness to spend necessary effort at work, which are seen at the hallmarks of burnout [31,32]. Therefore classified as secondary symptoms, they may also appear in other physical and mental disorders, such as hyperthyroidism, cancer, mood disorder, or anxiety disorder and depression [7,33]. The secondary symptoms that are evaluated through the BAT-S scale in this paper assess only two secondary symptoms (psychological distress and psychosomatic complaints) and do not include a subscale for depressed mood, as this aspect of well-being has already been effectively operationalized in several other questionnaires, such as the Four-Dimensional Symptom Questionnaire (4DSQ) [34]; therefore, there was no specific need to develop a new scale [7].

Evidence indicates that work-related stressful conditions encompass job stress, employment status, job insecurity, and imbalance between work and family life. Authors emphasized that job stress models and unstable employment conditions have major implications for both clinical and research contexts in psychosomatic medicine [35]. Work-related conditions consistently emerge as the most frequent stressors experienced by individuals, followed by health and financial problems, all associated with adverse physical and mental outcomes such as cardiovascular diseases and insomnia [33].

Extensive research has shown that psychosocial aspects of the working environment are strongly linked to adult health outcomes [35,36], particularly in the early manifestations of psychosomatic complaints and psychological distress, which overlap with symptoms commonly described in depression and burnout [37,38]. This underlines the importance of incorporating complementary tools for assessing burnout in occupational contexts and understanding burnout severity by capturing its consequences alongside its core manifestations. Therefore, secondary symptoms reflect a broader impact of prolonged work-related stressors on psychological distress and psychosomatic functioning and are especially relevant because they frequently represent the reason individuals seek professional help [7].

In the cross-national validation of the BAT, secondary symptoms correlated strongly with primary symptoms [10,21,29]. Supporting this interpretation, structural models indicate that secondary symptoms are best represented as correlating with primary burnout symptoms rather than as part of the latent core construct [6,7]. Furthermore, the Lithuanian validation study [21] confirmed the two-factor structure of secondary symptoms (psychological distress and psychosomatic complaints), demonstrating a significant difference (*p* < 0.001) between the unidimensional and two-factor models. The CFA showed a strong correlation between the two factors (r = 0.72) and confirmed they are positively associated with core burnout symptoms. Overall, these findings support the rationale for testing this two-factor structure in the present study’s confirmatory analysis of secondary symptoms.

Theoretically, distinguishing psychological distress from psychosomatic complaints reflects the multidimensional progression of burnout, whereby prolonged exposure to chronic work stress initially impacts emotional and cognitive functioning (e.g., irritability, concentration difficulties), which may subsequently evolve into physical manifestations (e.g., headaches, sleep disturbances) [35]. This cascading process underscores that psychological and psychosomatic complaints, while strongly associated, are not redundant dimensions but complementary indicators of the broader syndrome. Practically, this differentiation enhances the diagnostic and preventive utility of the BAT-S, as it allows practitioners to identify whether secondary symptoms manifest primarily in the psychological or psychosomatic domain, thereby informing targeted interventions and monitoring strategies [39,40]. The findings from the original BAT research also conclude with a recommendation for the application of the 6-item depression subscale from the Four-Dimensional Symptom Questionnaire (4-DSQ) [7,34].

The present study aims to assess psychometric evidence for the Brazilian Portuguese version of the Burnout Assessment Tool for Secondary Symptoms (BAT Brazil-S), testing the hypothesized models—specifically, assessment of BAT-S dimensionality, internal consistency, and convergent validity in the Brazilian context. The following hypotheses were proposed: (1) It is expected that the hypothesized two-factor oblique structure of the BAT-S will demonstrate a satisfactory fit to the data collected in Brazil, supporting evidence of its validity. (2) Based on previous studies [7,9], the BAT-S dimensions (psychological distress and psychosomatic complaints) are anticipated to demonstrate adequate internal consistency estimates (≥0.80). Therefore, this study seeks to provide further reliability evidence for the Brazilian version. (3) Another relevant source of validity evidence lies in the association between secondary symptom scores and burnout core symptoms. It is hypothesized that BAT-S scores will show positive correlations with general burnout and its core dimensions.

## 2. Materials and Methods

### 2.1. Participants

A convenience sample of 1.750 professionals in Brazil was analyzed. The participants lived in the four Brazilian macroeconomic regions, with 78.5% in the Center-South, 17.6% in the Northeast, 2.7% in the Legal Amazon, and 1.2% in the administrative region (Federal District). The participants met the inclusion criteria with a work status that required them to perform paid occupational activities autonomously or in an organization at the time of the research. At the time of the survey, the sample was composed of 11% white collar workers and 48.4% blue collar workers, and 26.5% were health workers. The majority (50.4%) had a postgraduate degree, with 24.9% having a college degree or ongoing and 24.7% having completed high school. The predominant salary range was between three to six salaries (40%). In the final sample, the mean age was 39 years (SD = 11), with 29% (n = 507) men and 71% (n = 1243) women.

### 2.2. Measures and Instruments

Sociodemographic and Labor Questionnaire: Created by the authors of the research with information about gender, age, education, and employment status.

The Burnout Assessment Tool, BAT-C and BAT-S: Burnout levels and their dimensions were assessed using the BAT developed and adapted to the Brazilian context [6,11]. The BAT-C scale (short version) for the work context consists of 12 items, which assess symptoms of exhaustion (3 items), mental distance (3 items), cognitive impairment (3 items), and emotional impairment (3 items). The BAT-S scale comprises 10 items designed to assess the two secondary dimensions of burnout symptoms, which are operationalized through the subscales of psychological distress or PD (5 items) and psychosomatic complaints or PC (5 items). BAT-C and BAT-S are rated on a five-point Likert scale ranging from never (1) to always (5). The adaptation and validation conducted, provided strong evidence for the psychometric adequacy of BAT-12 in Brazil [11], with a hierarchical structure of four first-order dimensions (exhaustion, mental distance, emotional impairment, and cognitive impairment) that load onto a second-order factor that assesses burnout. The reliability indices of the BAT-12 scale were satisfactory (α = 0.91, ω = 0.91, and C. R. = 0.90). The scale also showed satisfactory goodness-of-fit indexes (CFI = 0.984, TLI = 0.978 and RMSEA = 0.067 [90%, 0.060–0.073]). The global score can be evaluated at four levels of burnout—low (<25th percentile), moderate (25th percentile to 75th percentile), high (75th percentile to 95th percentile), and very high (>95th percentile)—based on the average score of participants’ responses [7,41].

### 2.3. Data Collection

The survey was administered between 2019 and 2024, with participants completing the instruments via an online platform and in person. Then, participants answered the sociodemographic questionnaire, BAT-C and BAT-S (Appendix A Table A1). The survey took approximately 15 min to complete. After excluding participants with invalid or incomplete information on BAT-C or BAT-S and univariate or multivariate outliers, the final analytic sample comprised 1750 participants. Participants, whether from in-person or online data collection, accessed the instruments after expressing their consent to participate in the research by agreeing to the Informed Consent Form (ICF) for in-person collection and the Online Consent Form Registration (RCEO) for online collection. These terms ensured the confidentiality and anonymity of the participants’ identities and information, detailing the research’s significance and objectives.

### 2.4. Ethical Considerations

The research project was submitted for evaluation by the Research Ethics Committee (CEP) in the university where it was developed in strict compliance with the applicable norms, guidelines, and ethical recommendations. Participants were invited to take part in the study voluntarily. Those who consented to participate completed the instrument after providing their agreement through the online Informed Consent Form. All collected material is for the exclusive use of the research team and will be used solely for the purpose of providing data for conducting the research itself and any resulting publications. Both in the research and in the production of the article, the confidentiality of the data and information that could identify participants will be ensured. The data collected will not be sold or disclosed in any way that could harm the participants and will be stored for at least five years, after which they may be destroyed.

### 2.5. Data Analysis

Evidence of validity for the internal structure of the Brazilian version of the BAT was examined using the total sample (N = 1750), randomly divided into two equal subsamples: one for exploratory analyses (n = 875) and another for confirmatory analyses (n = 875). Given that maximum likelihood (ML) estimation was employed and the items assess symptom-focused experiences, we examined univariate normality through skewness and kurtosis statistics. Reliability and external validity were evaluated with the full sample.

Exploratory Graph Analysis (EGA) [42] was conducted on the first subsample to identify the dimensional structure. EGA estimates partial correlations among items using graphical LASSO (λ = 0.10) with model selection based on the extended Bayesian information criterion (EBIC; γ = 0.50) [43]. The walktrap algorithm [42] was applied to detect clusters, and item stability was assessed via bootstrapping with values ≥ 0.75 considered acceptable [44]. Analyses were conducted in R version 4.5.2 with the EGAnet package [45].

Confirmatory Factor Analysis (CFA) was performed on the second subsample, testing three models: (a) the EGA-derived structure, (b) a two-factor oblique model, and (c) a bi-factor model with two oblique factors. Models were estimated with the ULSMV estimator, appropriate for ordinal data, using BFGS optimization. ULSMV has been shown to provide robust parameter estimates and χ^2^ statistics [46,47,48]. Model fit was evaluated using χ^2^, CFI, TLI, RMSEA, and SRMR, with cutoffs of CFI/TLI ≥ 0.95, RMSEA ≤ 0.06, and SRMR ≤ 0.08 [49]. CFA analyses were conducted in R using lavaan [50].

Differential Item Functioning (DIF) was tested across age, gender, and educational level using ordinal logistic regression for polytomous items [51]. Three nested models were compared: (a) a baseline model including only the latent trait (θ), (b) a uniform DIF model adding the group variable, and (c) a non-uniform DIF model including the interaction between group and trait. DIF was flagged when the likelihood ratio test was significant (*p* < 0.01) and ΔR^2^ ≥ 0.035 [52]. Analyses were performed with the lordif package [51].

Finally, Structural Equation Modeling (SEM) was used to examine associations between BAT dimensions (exhaustion, mental Distance, cognitive impairment and emotional impairment) and the validated constructs. SEM was conducted in R with lavaan [50] using the ULSMV estimator. Model fit was evaluated via RMSEA, SRMR, CFI, and TLI [49]. Correlations were interpreted according to magnitude, direction, and statistical significance (*p* < 0.05).

## 3. Results

First, the skewness and kurtosis of the score distributions of the BAT items were examined. As shown in Table 1, skewness values ranged from −0.314 to 1.058, and kurtosis values ranged from −1.043 to 0.313. All values were within commonly accepted thresholds, according to the criteria of Weston & Gore (2006) (skewness < 3 kurtosis < 7), indicating no substantial deviations from normality [53].

**Figure 1:**
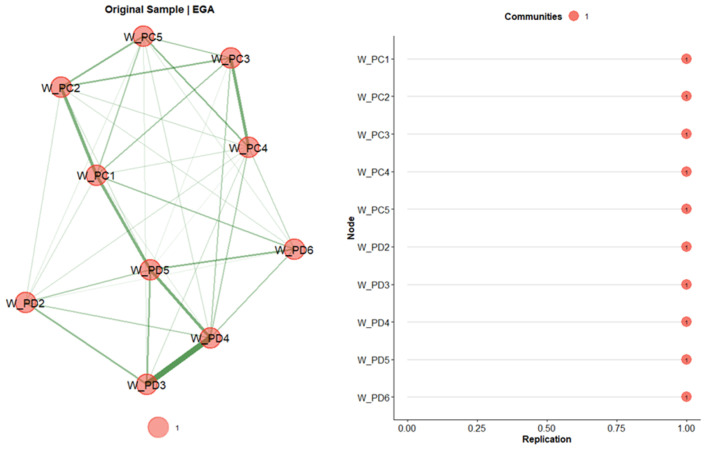
Results of the Exploratory Graph Analysis with bootstrap and item stability. Note. W_PC1 to W_PC5 represent items from the psychosomatic complaints scale: 1 = palpitations or chest pain; 2 = stomach and/or intestinal complaints; 3 = headaches; 4 = muscle pain; 5 = getting sick often. W_PD2 to W_PD6 represent items from the psychological distress scale: 2 = trouble falling or staying asleep; 3 = worry; 4 = tense and stressed; 5 = anxious and/or suffer from panic attacks; 6 = noise and crowds are disturbing. The W_PD1 item was not considered as it was excluded in the original validation study. The green lines represents the correlations between the items. The bolder the stronger the correlation.

We conducted an Exploratory Graph Analysis (EGA) including only the secondary symptoms of the instrument (Figure 1). The community structure obtained from EGA revealed a single cohesive cluster, suggesting a unidimensional solution for BAT-S. Each item demonstrated a replication index of 1, indicating strong structural stability and consistent item contribution to a single latent factor.

Three alternative models for BAT-S were assessed through Confirmatory Factor Analysis (CFA): (1) unidimensional model, derived from the Exploratory Graphic Analysis (EGA), (2) two-factor oblique model (e.g., correlated) aligned with the conceptualization of burnout as a syndrome within the framework of the BAT [6], (3) bi-factor model with two oblique factors; according to previous validation studies that have tested alternative factorial models [21,29], such as the higher-order structure for the core symptoms, this selected model more precisely reflects the theorized structure of secondary symptoms in the BAT framework [7].

The unidimensional model exhibited the poorest fit (CFI = 0.951, TLI = 0.937, RMSEA = 0.115), in comparison to the other solutions. The two oblique-factor first-order factors showed an improvement across all indices (CFI = 0.972, TLI = 0.963, RMSEA = 0.088). Finally, the bi-factor model with two correlated factors demonstrated the best overall fit (CFI = 0.992, TLI = 0.985, RMSEA = 0.056), indicating superior representation of the data structure (Table 2).

Although the bi-factor solution provided the best global fit, it also absorbed a substantial portion of variance from the content-specific factors, thereby reducing their explanatory contribution of the latent dimensions that constitute BAT-S (Table 3), concluding with the selection of the two-factor oblique model as the most adequate solution. The factor loadings for the psychosomatic complaints scale indicated the items need to be interpreted with more parsimony than the psychological distress scale. For psychosomatic complaints, the item “I suffer from headaches” showed the lowest factorial loading that provides the least discrimination, whereas the item “I suffer from muscle pain, for example in the neck, shoulder or back” discriminates most effectively, reflecting a greater sensitivity of the item to differences in the subscale and more representativity of the latent trait. In the case of psychological distress, the item “Noise and crowds disturb me” with the lowest factor loading discriminates less, and the item “I feel tense and stressed” showed the highest factor loading in the scale, representing this latent trait and reflecting an item with higher sensitivity to differences in the latent trait [54].

The two oblique-factor first-order factors model exhibited adequate-to-good internal consistency (i.e., coefficients ≥ 0.80). Specifically, the psychological distress (PD) factor showed reliability coefficients of α = 0.88 and ω = 0.90, while the psychosomatic complaints (PC) factor demonstrated reliability coefficients of α = 0.85 and ω = 0.87, indicating satisfactory measurement precision for both constructs.

When conducting Differential Item Functioning (DIF) analysis with the full sample, all items were invariant (i.e., no item showed *p* < 0.01 combined with Nagelkerke’s pseudo R^2^ ≥ 0.035) across gender, age, and educational level (Table 4 and Table 5).

The Structural Equation Modeling (SEM) analysis with the measurement models (([0.049, 0.056]), CFI = 0.973, and TLI = 0.968) indicated that the specified model and factor correlations showed an adequate fit (SRMR = 0.034, RMSEA = 0.052 (90% CI adequately fit the data)). Exhaustion was strongly and positively correlated with mental distance (r = 0.714, *p* < 0.001) and cognitive emotional impairment (r = 0.576, *p* < 0.001). Mental distance was also positively correlated with cognitive emotional impairment (r = 0.679, *p* < 0.001). Regarding sociodemographic variables, no statistically significant differences were observed across BAT dimensions; therefore, these analyses were not included in the present manuscript and the results can be obtained by contacting the authors.

Regarding the scales validated in this study, psychosomatic complaints and psychological distress showed positive associations with all burnout symptoms. In particular, exhaustion emerged as the most strongly related symptom, presenting the highest correlations with both psychosomatic complaints (r = 0.661, *p* < 0.001) and psychological distress (r = 0.748, *p* < 0.001). In addition, both scales were positively correlated with mental distance and cognitive impairment and emotional impairment, with all remaining associations being moderate to strong (r > 0.47). Finally, psychosomatic complaints and psychological distress were strongly and significantly correlated with each other (r = 0.86, *p* < 0.001).

## 4. Discussion

The present study, as the first in Brazil, aimed to examine the psychometric properties of the Brazilian adaptation of the BAT-S, focusing on its structural validity, internal consistency, and its relations with burnout core symptoms. The findings provide robust support for the proposed hypotheses, reinforcing the adequacy of the BAT-S as an instrument for assessing secondary symptoms of burnout in the Brazilian occupational context, in line with the results shown by the studyin the Lithuanian context [21].

The dimensionality analysis represents a central contribution of this study, as it clarifies the factorial structure of the BAT-S. Consistent with H1 and based on the results from the Lithuanian validation study [21]), the hypothesized two-factor oblique model structure of the BAT-S demonstrated a satisfactory fit to the data collected in Brazil, supporting evidence of its validity. This result aligns with cross-cultural validations and demonstrates the flexibility of the BAT-S to capture nuanced symptom profiles across diverse contexts. It is important to note that unidimensional structure is discarded based on the evidence from the CFA and other studies [7,21]. The evidence from this study shows that the BAT-S scale should not be conceptualized as unidimensional, as its two factors represent complementary yet distinct manifestations of secondary symptoms which should be examined together rather than collapsed into a single general dimension. Understanding the differential role of these two secondary symptom domains could make a difference in burnout assessment and prevention [55]. The literature defines psychological distress as a series of psychophysiological and behavioral reactions that are nonspecific to any single pathology, encompassing anxious and depressive responses, irritability, cognitive decline, and sleep disturbances [56]. When left untreated, psychological distress may evolve into serious but reversible health conditions (e.g., hypertension, severe depression, alcoholism, burnout) [57], which are also associated with the emergence of psychosomatic complaints that, over time, may lead to irreversible outcomes such as permanent disability, premature death, or severe chronic cardiovascular and neuropsychiatric diseases [57]. Therefore, the early detection of secondary symptoms in organizational settings is critical to identifying health deterioration at its onset and enabling timely preventive interventions [58,59].

All items demonstrated adequate factor loadings, supporting the view of secondary symptoms as outcomes of prolonged exposure to work-related stressors rather than defining features of burnout [7]. The items “headaches” (psychosomatic complaints) and “noise and crowds are disturbing” (psychological distress) exhibited comparatively lower loadings. This pattern aligns with the Burnout Assessment Tool framework, as such symptoms are not specific to burnout but overlap with broader forms of psychological distress (e.g., anxiety and depression) [60]. Accordingly, their slightly lower loadings likely reflect shared variance with general distress constructs and their situational nature, rather than weaknesses in the underlying factor structure [7]. In both psychosomatic complaints and psychological distress, items with higher factor loadings (the item “muscle pain” for psychosomatic complaints and “tense and stressed” for psychological distress) demonstrated greater representativity of the latent trait, respectively, whereas items related to headaches or sensitivity to noise (the item “headaches” for psychosomatic complaints and “noise and crowds are disturbing” for psychological distress) showed weaker representation of the dimensions. For the higher-factor-loading items, this reflects a more global physiological activation (e.g., generalized muscle tension or psychological tension and stress) that makes sense as more stable allostatic load processes that accumulate over time, making them stronger indicators of chronic dysregulation. In contrast, items with lower factorial loadings are more situational or stimulus-dependent reactions capturing fluctuations that may be reactive rather than baseline-defining, and therefore load on less central variance of the latent construct [61]. From a practical perspective, in assessment, this distinction is critical, as higher-loading items may serve as earlier and more sensitive markers of chronic health deterioration, whereas lower-loading items may reflect context-specific discomfort that is less predictive of long-term impairment.

Consistent with H2, the BAT-S demonstrated excellent internal consistency, with Cronbach’s alpha and McDonald’s omega coefficients exceeding the 0.80 threshold for both subscales [62]. These results confirm that the BAT-S provides reliable and stable measurement of secondary symptoms in Brazilian professionals, supporting its utility in both research and clinical applications.

In support of H3, the BAT-S showed strong positive correlations with the core dimensions of burnout, mental exhaustion, mental distancing, cognitive impairment, and emotional impairment. Mental exhaustion exhibited the strongest associations with psychological distress and psychosomatic complaints, suggesting that exhaustion plays a central role in triggering secondary symptoms [63]. This is consistent with theoretical models positing that secondary symptoms are downstream consequences of primary burnout symptoms [7]. From a practical perspective, the validation of the BAT-S in Brazil enhances its applicability for occupational health monitoring and intervention. Identifying secondary symptoms, psychological distress, and psychosomatic complaints can help individuals to identify the initial stages of stress impact on psychological distress and psychosomatic complaints, facilitating timely intervention [64,65].

The validation of the BAT-S within the Brazilian context represents a significant step toward standardized burnout assessment in both research and applied settings. By enabling the evaluation of secondary symptoms alongside core dimensions, the BAT supports a more comprehensive conceptualization of burnout, facilitating early detection, accurate diagnosis, and personalized intervention strategies. This approach aligns with international recommendations for multidimensional assessment and strengthens occupational health policies focused on prevention and well-being promotion [66,67].

## 5. Conclusions

In conclusion, the Brazilian BAT-S demonstrates satisfactory psychometric properties and theoretical coherence, confirming its utility for research, clinical practice, and occupational health management. This study’s strengths include its large sample and the use of robust psychometric methods (EGA and CFA) to examine structural validity. However, some limitations warrant consideration; the cross-sectional design precludes causal inference or conclusions about the temporal development of symptoms. Additionally, the sample was predominantly female (71%), which may limit the generalizability of the results. Furthermore, the sample was relatively homogeneous which limited the between-group comparison and resulted in non-significant differences, thereby reducing the relevance of these analyses for the present study. By bridging conceptual gaps and addressing previous measurement limitations, the BAT-S contributes to advancing burnout diagnosis, prevention, and intervention in both national and international contexts. It would also be relevant to investigate the association between secondary burnout symptoms and individuals’ illness attitudes, as this may contribute to a more refined understanding of the nomological network and underlying mechanisms of the burnout construct.

Future research should investigate the longitudinal stability of the BAT-S structure and its predictive validity regarding health and performance outcomes. Moreover, integrating psychometric data with biomarkers (e.g., cortisol levels) and objective behavioral indicators could enhance the clinical utility and diagnostic precision of the BAT-S. Cross-cultural comparisons should also be conducted to advance the global standardization of burnout assessment. Further research is needed to investigate the role of the BAT-S as a complementary tool with BAT-C in clinical practice.

## Figures and Tables

**Table 1:** Descriptive statistics for the BAT-S items.

Items	Min–Max	Mean	SD	Skewness	Kurtosis
PD2. Trouble falling or staying asleep	1–5	2.630	1.293	0.310	−1.043
PD3. Worry	1–5	3.481	1.100	−0.314	−0.655
PD4. Tense and stressed	1–5	3.288	1.083	−0.147	−0.664
PD5. Anxious and/or suffer from panic attacks	1–5	2.454	1.267	0.388	−0.968
PD6. Noise and crowds are disturbing	1–5	2.702	1.253	0.252	−0.904
PC1. Palpitations or chest pain	1–5	1.899	1.102	1.058	0.260
PC2. Stomach and/or intestinal complaints	1–5	2.317	1.196	0.519	−0.736
PC3. Headaches	1–5	2.774	1.170	0.182	−0.822
PC4. Muscle pain	1–5	3.287	1.186	−0.221	−0.841
PC5. Getting sick often	1–5	2.237	0.989	0.777	0.313

Abbreviations: Min–Max, minimum and maximum value; SD, standard deviation.

**Table 2:** Fit indices for the models tested.

Model	*χ*^2^ (gl) [*p*-Value]	CFI	TLI	RMSEA [IC 90%]	SRMR
Unidimensional (EGA)	439.718 (35) [*p* < 0.001]	0.951	0.937	0.115 [0.106–0.125]	0.055
Two-Factor Oblique	262.529 (34) [*p* < 0.001]	0.972	0.963	0.088 [0.078–0.098]	0.041
Bi-factor with Two Oblique Factors	90.354 (24) [*p* < 0.001]	0.992	0.985	0.056 [0.044–0.069]	0.023

**Table 3:** Factor loadings for the tested models.

Item	Model					
	Unidimensional	Two-Factor Oblique		Bi-Factor with Two Oblique Factors		
PC1. Palpitations or chest pain	0.724	0.755		0.877	0.015	
PC2. Stomach and/or intestinal complaints	0.699	0.733		0.658	0.319	
PC3. Headaches	0.663	0.698		0.514	0.540	
PC4. Muscle pain	0.722	0.758		0.529	0.668	
PC5. Getting sick often	0.670	0.704		0.598	0.371	
PD2. Trouble falling or staying asleep	0.690		0.711	0.581		0.394
PD3. Worry	0.761		0.790	0.535		0.654
PD4. Tense and stressed	0.844		0.875	0.608		0.700
PD5. Anxious and/or suffer from panic attacks	0.842		0.871	0.754		0.409
PD6. Noise and crowds are disturbing	0.677		0.696	0.602		0.329

**Table 4:** DIF statistics for items in the psychosomatic complaints scale.

Item	Group	*p*-Value			Pseudo R^2^ Nagelkerke		
		Model 1 vs. Model 2	Model 1 vs. Model 3	Model 2 vs. Model 3	Model 1 vs. Model 2	Model 1 vs. Model 3	Model 2 vs. Model 3
Palpitations or chest pain	Sex	0.0038	0.0139	0.6818	0.0026	0.0027	0.0001
Stomach and/or intestinal complaints	Sex	0.8816	0.475	0.2259	0	0.0004	0.0004
Headaches	Sex	0.5449	0.6471	0.4778	0.0001	0.0002	0.0001
Muscle pain	Sex	0.8519	0.224	0.0855	0	0.0006	0.0006
Getting sick often	Sex	0.0033	0.0123	0.6896	0.0024	0.0024	0
Palpitations or chest pain	Age	0.7037	0.0699	0.0186	0.0002	0.0027	0.0025
Stomach and/or intestinal complaints	Age	0.3612	0.4687	0.4668	0.0005	0.0009	0.0004
Headaches	Age	0.0001	0.0002	0.334	0.0042	0.0047	0.0005
Muscle pain	Age	0.0049	0.0259	0.8123	0.0022	0.0023	0.0001
Getting sick often	Age	0.1462	0.3022	0.6028	0.0011	0.0014	0.0003
Palpitations or chest pain	Education	0.0278	0.0782	0.5408	0.0027	0.0032	0.0005
Stomach and/or intestinal complaints	Education	0.8104	0.4145	0.1723	0.0001	0.0012	0.0011
Headaches	Education	0.1941	0.0382	0.0324	0.0009	0.0028	0.0019
Muscle pain	Education	0.0054	0.0222	0.6156	0.0027	0.003	0.0002
Getting sick often	Education	0.2161	0.4865	0.8273	0.0009	0.0011	0.0001

**Table 5:** DIF statistics for items in the psychological distress scale.

Item	Group	*p*-Value			Pseudo R^2^ Nagelkerke		
		Model 1 vs. Model 2	Model 1 vs. Model 3	Model 2 vs. Model 3	Model 1 vs. Model 2	Model 1 vs. Model 3	Model 2 vs. Model 3
Trouble falling or staying asleep	Sex	0.6072	0.7703	0.6117	0.0001	0.0002	0.0001
Worry	Sex	0.1364	0.3227	0.8339	0.0003	0.0003	0
Tense and stressed	Sex	0.0898	0.2215	0.7112	0.0003	0.0003	0
Anxious and/or suffer from panic attacks	Sex	0.0291	0.0758	0.5277	0.0009	0.001	0.0001
Noise and crowds are disturbing	Sex	0.0346	0.1064	0.8983	0.0016	0.0016	0
Trouble falling or staying asleep	Age	0	0	0.3356	0.0174	0.0181	0.0007
Worry	Age	0.6434	0.9269	0.9992	0.0001	0.0001	0
Tense and stressed	Age	0.0001	0.0008	0.5328	0.0016	0.0017	0.0001
Anxious and/or suffer from panic attacks	Age	0.0283	0.0236	0.1253	0.0013	0.0021	0.0008
Noise and crowds are disturbing	Age	0.0123	0.0553	0.8005	0.0031	0.0033	0.0002
Trouble falling or staying asleep	Education	0.43	0.5482	0.5041	0.0007	0.0013	0.0006
Worry	Education	0.9241	0.27	0.0815	0	0.0009	0.0009
Tense and stressed	Education	0.7487	0.9274	0.8603	0.0001	0.0001	0
Anxious and/or suffer from panic attacks	Education	0.3853	0.4732	0.4441	0.0005	0.0009	0.0004
Noise and crowds are disturbing	Education	0.8451	0.101	0.0245	0.0001	0.0033	0.0031

## Data Availability

The data presented in this study are available on request from the corresponding author.

## Appendix A

**Table A1:** Portuguese version of BAT-S.

	Nunca (Never)	Raramente (Rarely)	Algumas Vezes (Sometimes)	Com Frequência (Frequently)	Sempre (Always)
Queixas Psicológicas	**1**	**2**	**3**	**4**	**5**
1. Tenho dificuldades em adormecer ou ficar dormindo					
2. Tendo a ser preocupado					
3. Sinto-me tenso e estressado					
4. Sinto-me ansioso e/ou sofro de ataques de pânico					
5. Barulho e multidões me perturbam					
Queixas Psicossomáticas	**1**	**2**	**3**	**4**	**5**
1. Sofro de palpitações ou dores no peito					
2. Sofro de dores no estômago e/ou intestinais					
3. Sofro de dores de cabeça					
4. Sofro de dores musculares, como, no pescoço, ombro ou costas					
5. Fico doente muitas vezes					
